# Effectiveness of health ecology model-based pulmonary rehabilitation for acute exacerbation of chronic obstructive pulmonary disease: a preliminary study

**DOI:** 10.3389/fmed.2026.1742282

**Published:** 2026-03-19

**Authors:** Ying Xu, Guiying Gan, Fei Ding, Hong Wu, Huamei Zheng

**Affiliations:** Department of Respiratory and Critical Care Medicine, Zhujiang Hospital of Southern Medical University, Guangzhou, China

**Keywords:** comprehensive intervention, effectiveness assessment, health ecology, health education, pulmonary rehabilitation

## Abstract

**Background:**

Acute exacerbation of chronic obstructive pulmonary disease (AECOPD) significantly impairs patients’ quality of life and survival. While pulmonary rehabilitation (PR) is well-established for stable COPD, evidence for its effectiveness during and after hospitalization for AECOPD remains limited. This study aimed to evaluate the effectiveness of a health ecology model (HEM)-based multidimensional PR intervention compared to conventional care in patients with AECOPD.

**Methods:**

This prospective randomized controlled trial enrolled 46 AECOPD patients who, after individual matching, were randomly allocated to either an experimental group or a control group. The experimental group received conventional care supplemented by an HEM-based intervention comprising physiological (assisted sputum removal, progressive breathing exercises, physical training, home-based PR guidance), psychological (peer and caregiver support), and environmental (temperature and humidity regulation) components. The control group received conventional care only, including health education, oxygen therapy, and breathing training during hospitalization. The intervention period spanned from the second day after admission to discharge, with FEV1% reassessed at 8 weeks post-discharge. Outcomes included predicted forced expiratory volume in one second (FEV1%), 6-min walk test (6MWT), modified Medical Research Council dyspnea scale (mMRC), and COPD Assessment Test (CAT). Between-group and within-group differences were analyzed using Student’s t-test, with statistical significance set at *p* < 0.05.

**Results:**

The experimental group demonstrated significantly greater improvements than the control group in mMRC scores (mean reduction 1.48 vs. 0.87 points, *p* = 0.001) and FEV1% (mean increase 6.72% vs. -0.02%, *p* = 0.025). Both groups showed significant within-group improvements in CAT scores and 6MWT distance, but no significant between-group differences were observed for these outcomes. All improvements in the experimental group exceeded established minimal clinically important differences (MCID).

**Conclusion:**

The HEM-based multidimensional PR intervention produced clinically meaningful and statistically significant improvements in lung function and dyspnea in patients hospitalized with AECOPD. These findings support integrating multi-level interventions—including physiological, psychological, and environmental components—into standard care to optimize outcomes in domains that show limited response to conventional rehabilitation alone.

## 1 Introduction

Chronic obstructive pulmonary disease (COPD) is the third leading cause of morbidity and mortality worldwide, with patients experiencing 0.5 to 3.5 acute exacerbations per year on average (1, 2). The acute exacerbation of COPD (AECOPD) is characterized by a rapid deterioration of chronic obstructive pulmonary disease (COPD) symptoms, often leading to accelerated lung function decline, quality of life impairment, and increased mortality (3). AECOPD should be diagnosed when patients exhibit worsening dyspnea, increased cough, and changes in the nature of the sputum in <14 days (4). It is a dangerous stage and key event in the course of COPD, as its deterioration and frequency seriously affect the quality of life of patients and shorten their survival (2). Approximately 13% of patients with AECOPD visited the hospital more than six times within a year, and half of them had a hospitalization interval of less than 6 months (5). The clinical guidelines by the European Respiratory Society (ERS) and the American Thoracic Society (ATS) emphasize that implementing pulmonary rehabilitation (PR) during hospitalization can effectively enhance patients’ physical well-being. Numerous studies have established the safety and efficacy of PR in both stable and acute COPD; however, further improvement in certain clinical outcomes remains a challenge, and the optimal strategies to enhance its therapeutic impact are yet to be determined. A systematic review of 42 trials involving 2,150 stable COPD patients confirmed that while PR significantly improves dyspnea, quality of life, and exercise capacity, but it fails to enhance daily physical activity, and substantial heterogeneity in intervention protocols persists (6). Machado et al. demonstrated that a 3-week home-based PR program was safe and feasible for patients recovering from moderate AECOPD. Compared to standard pharmacotherapy alone, it significantly improved symptoms and muscle strength (7). However, COPD patients who experience exacerbations exhibit less favorable responses to PR, demonstrating significantly smaller gains in physical performance, respiratory muscle strength, and lung function than their counterparts without exacerbations (8).

Health is often conceptualized in terms of fixed biological parameters, while the complex interplay of environmental and cultural factors receives less attention. The ecological perspective on health was significantly advanced by American psychologist Urie Bronfenbrenner, who proposed in 1977 that human development and health are influenced by environmental factors across four dimensions: the individual (microsystem), interpersonal (mesosystem), community (exosystem), and society (macrosystem), laying the foundation for the theory of health ecology (9). Furthermore, McLeroy et al. integrated ecological theory into health promotion, emphasizing the causality between individuals and their environment (10). The combined effects of individual physiology, personality characteristics, behavioral patterns, surrounding natural and social environments, and health services provided were highlighted for health improvement. These factors were subsequently classified by researchers into various categories or different stages to illustrate how the environment affected health (11). The health ecology model (HEM) was thus established and gained increasing recognition and employment in recent years. In the domain of PR, though breathing exercise and physical activity have been extensively proven to effectively improve patients’ lung function, their adherence has fallen short of expectations. As a natural response, avoidance of exercise and physical activity leads to deleterious effects outside the lungs and perhaps accelerates the inflammatory cascade in the lungs of patients (12). Multi- and interdisciplinary collaboration was considered to aid in the translation of increased exercise capacity to greater participation in physical activities (13). However, such collaboration was usually limited to healthcare workers of various specialties in the past. The theoretical framework of HEM suggests that the PR program should aim to improve patients’ own conditions (including physiological, psychological, cognitive, and behavioral aspects), medical services, social interactions and support, and policy assistance. Similar applications of HEM have achieved proactive impacts in the intervention and management of hypertension, diabetes, heart failure, and depression through stimulating patients’ self-efficacy and forming all-around support (14). While emerging research has adopted ecological perspectives to investigate psychological outcomes in AECOPD patients, such as intolerance of uncertainty influenced by multi-level factors including psychological flexibility, health literacy, and social support (15), and protocol studies have begun exploring the impact of urban environments on physical activity in COPD, these investigations remain fragmented and have not yet translated into integrated, HEM-guided rehabilitation programs. To date, few published studies have operationalized the comprehensive HEM framework to guide the design and implementation of pulmonary rehabilitation for patients with COPD, particularly during acute exacerbation.

The study hypothesized that HEM-based multidimensional approach would yield superior improvements in clinical outcomes compared to conventional care alone, with the integration of psychological and environmental components specifically contributing to enhancements in outcomes that typically show limited response to conventional PR, such as lung function and dyspnea relief. In the current study, therefore, evidence-based measures of different dimensions were initially integrated into the PR program. Lim et al. argued that financial difficulty was a primary reason for the inability to complete PR in patients with COPD; hence, medical insurance staff would be invited as a policy speaker to ease patients’ economic burden in the study (16). Several studies have shown that patients seek knowledge from their support groups and feel empowered by sharing and receiving knowledge and experiences (17, 18). Support from ward mates with AECOPD, caregivers, and direct relatives will be highlighted for enhancing self-efficacy in long-term PR. Additionally, home-based management, as a form of continuing healthcare for PR, including portable equipment instructions, breathing exercises, and physical activity supervision, will be implemented to strengthen expected effects. It will also be explored to regulate the indoor microclimate in both wards and houses.

## 2 Methods

### 2.1 Study design

This study was designed as a prospective, parallel-group randomized controlled trial, and implemented at a provincial hospital in southern China from January 2023 to June 2024, primarily employing well-defined, established intervention measures. Patients in the control and experimental groups were assigned to separate wards located at a considerable distance from each other to ensure mutual unawareness and prevent contamination. Due to the nature of the rehabilitation intervention, blinding of participants and the nurses delivering the intervention was not feasible; therefore, this was an open-label trial with respect to intervention delivery. However, outcome assessors were blinded to group allocation. All post-intervention assessments (6MWT, spirometry, mMRC, and CAT) were conducted by trained research assistants who were not involved in intervention delivery and had no access to group assignment information. Participants were instructed not to disclose their group allocation to the assessors.

This study was approved by the Ethics Committee of Zhujiang Hospital of Southern Medical University (No. 2022-KY-125-02) and conducted in accordance with the principles of the Declaration of Helsinki. Written informed consent was obtained from all participants prior to enrollment. Potential participants were approached by research nurses during the first 24 h of hospitalization. They were provided with detailed verbal and written information about the study purpose, procedures, potential benefits and risks, confidentiality protections, and their right to withdraw at any time without affecting their medical care. A minimum of 12 h was allowed for consideration before consent was sought. Data collection was performed by trained research assistants at two time points: within 48 h of admission (baseline) and at discharge (post-intervention). FEV1% was additionally measured at 8 weeks post-discharge during a scheduled outpatient follow-up visit. All data were recorded on standardized case report forms and subsequently entered into a secure electronic database with double-data entry verification.

### 2.2 Participants

Hospitalized patients during AECOPD were selected based on Guidelines for the Diagnosis and Management of Chronic Obstructive Pulmonary Disease (revised version 2021) by the Chinese Thoracic Society (19). Patients were recruited if they met all of the following inclusion criteria: (1) age between 40 and 79 years, (2) diagnosed with AECOPD, (3) no reading or communication disorders. Patients will be excluded once any of the following exclusion criteria appear: (1) having severe cardiac or pulmonary dysfunction, (2) cognitive impairment, (3) affected by the rehabilitation exercises due to movement system disorders, (4) refusal to participate in the study. If a dropout occurs in either member of a pair, the pair will be excluded from the formal analysis. Participants were matched 1:1 by age (±2 years), sex, current smoking status, and pulmonary dysfunction grade. An independent statistician generated a computer-randomized allocation sequence with a 1:1 ratio, which was concealed in sequentially numbered, opaque, sealed envelopes. After a matched pair was deemed eligible, the research nurse opened the next envelope in the participant’s presence to reveal group assignment. This procedure ensured that recruitment and matching personnel remained blinded to allocation until assignment, thereby minimizing selection bias.

### 2.3 Sample size estimation

Sample size was calculated using G*Power software (version 3.1.9.7). Based on a previous pilot study examining PR outcomes in AECOPD patients, which reported a moderate effect size for the primary outcome of FEV1% improvement, we determined that a minimum of 21 participants per group would be required to detect a significant difference with 80% power and a two-tailed *α* of 0.05. To account for an anticipated dropout rate of approximately 10% based on similar rehabilitation studies in acutely ill populations, we aimed to recruit 26 participants per group. Ultimately, 58 participants were enrolled to ensure adequate power after potential exclusions, and 46 completed the study.

### 2.4 Intervention

PR plans were formulated based on a joint assessment by respiratory physicians, rehabilitation therapists, and specialist nurses within two days of admission, and executed by trained nurses under professional guidance. Prior to study initiation, all nurses responsible for intervention delivery (*n* = 6) underwent a standardized 8-h training program conducted by the research team. The training covered: (1) theoretical background of HEM and PR principles; (2) demonstration and supervised practice of all physiological interventions including ACBT, high-frequency chest wall oscillation, progressive breathing training, and physical exercises; (3) communication skills for providing psychological support; (4) proper use and calibration of environmental monitoring equipment; and (5) standardized documentation procedures. Competency was assessed through direct observation and return demonstration, with a minimum passing score of 90% required before nurses could deliver interventions independently. Refresher training was provided quarterly throughout the study period to maintain intervention fidelity.

#### 2.4.1 Intervention of the control group

Control group patients received conventional interventions, including: (1) manuals of COPD control, smoking cessation guidance, and standardized admission education carried out by the responsible nurse; (2) low-flow oxygen inhalation therapy at 1–3 L/min and ultrasonic nebulization inhalation three times a day; (3) 10- to 20- min’ training of pursed-lip breathing (PLB) combined with diaphragmatic breathing (DB) twice a day.

#### 2.4.2 Intervention of the experimental group

The interventions for the experimental group were conducted using HEM-based multidimensional measures in addition to those in the control group, which included assisted sputum removal, progressive breathing training, physical exercises, home-based PR guidance (physiological), peer and caregiver support (psychological), and indoor microclimate regulation (environmental).

##### 2.4.2.1 Physiological interventions

###### 2.4.2.1.1 Assisted sputum removal

The active cyclic breathing technique (ACBT) combined with high-frequency chest wall oscillation for sputum removal, was used for airway clearance twice daily. The former lasted for 20 min each time and the latter for 10 min, with a frequency of 8–14 Hz and a pressure of 2–4 kPa.

###### 2.4.2.1.2 Progressive breathing training and physical exercises

Breathing training was implemented with PLB plus DB following an exhalation-to-inhalation ratio of 1:3 for 20 min twice a day, and resistance-relaxation cycle breathing exercises, which involved a set of movements of upper limb abduction and trunk lateral flexion once a day. A sitting-position electric leg trainer and hand-muscle developer for the lower and upper limbs were used for progressive physical training. The intensity of the limbs started with 70% of the maximum oxygen uptake at a low speed using the 0.5th gear, and the resistance parameters of the trainer were dynamically adjusted according to the patient’s endurance. It was conducted for a duration of 10 to 20 min, once a day. Upper-limb exercises were performed with the shoulder flexed at 60 to 90 degrees, starting with the initial weight based on the individual’s grip strength test. Participants performed progressively increasing exercise sets (1–4 sets) based on individual endurance, with alternating left and right repetitions within each set. Pre-training and dynamic physical monitoring, including heart rate and oxygen saturation, were performed to ensure participants’ health and safety.

###### 2.4.2.1.3 Home-based PR guidance

Patient received individually tailored health education and guidance once a week after their discharge, including proper operation of inhalation devices and non-invasive ventilators, family-based standardized oxygen therapy, and the recognition and handling of acute exacerbation warnings. This continuing care, which lasted 8 weeks, was accomplished through the collaboration of patients, caregivers, and researchers. To monitor and enhance adherence to the home-based PR program, multiple strategies were implemented: (1) patients were provided with a structured exercise diary to record daily breathing exercises, physical activities, and any symptoms or concerns; (2) weekly telephone follow-ups were conducted by research nurses to review diary entries, address questions, and provide reinforcement; (3) a dedicated WeChat group was created for each patient, enabling real-time communication with the research team and allowing patients to share progress photos or videos of their exercises; (4) caregivers were trained to supervise and encourage daily exercise completion and to document any deviations from the prescribed regimen. Adherence was defined as completing ≥80% of prescribed exercise sessions per week, and patients falling below this threshold received additional motivational interviewing during telephone follow-ups.

##### 2.4.2.2 Psychological interventions

###### 2.4.2.2.1 Economic assistance policy propaganda

For relieving patients’ stress and motivating their self-efficacy, medical insurance staff were invited to propagandize the preferential treatment during hospitalization and after discharge, such as a lower deductible and higher reimbursement for certain chronic and special diseases (including COPD) according to the current medical insurance policy in China. Meanwhile, additional special medical aid was provided to further alleviate the burden for those with poor economic conditions.

###### 2.4.2.2.2 Peer and caregiver support

A peer support group was established, and its opinion leader was encouraged and cultivated in the experimental group. Patients were invited to participate in the offline sharing session in a conference room once a week during their hospitalization. The schedule was formulated by researchers to ensure each patient participated in at least one session. Those who have recently achieved significant recovery shared their experiences with others. Meanwhile, a WeChat group was created to facilitate online communication among patients and mutual encouragement.

##### 2.4.2.3 Environmental interventions

The temperature and humidity in the patients’ wards and bedrooms were monitored and regulated, keeping the indoor temperature at 22–25 degrees Celsius and the humidity between 30 and 80% in winter, and the indoor temperature at 23–28 degrees Celsius and the humidity between 40 and 60% in summer. Their bedrooms were also moved to rooms far away from the kitchen, and air purifiers were installed, or the rooms were ventilated for at least 4 h every day.

##### 2.4.2.4 Safety monitoring and risk management

To ensure participant safety throughout the intervention, comprehensive risk management procedures were implemented. Prior to each exercise session, participants underwent pre-exercise assessment including heart rate, blood pressure, oxygen saturation (SpO₂), and symptom evaluation (dyspnea, chest pain, dizziness). Exercise was postponed if any of the following criteria were met: resting heart rate >120 bpm, systolic blood pressure >180 mmHg or <90 mmHg, SpO₂ < 88% on room air, acute chest pain, uncontrolled arrhythmias, or fever >38 °C (20). During exercise sessions, continuous pulse oximetry and heart rate monitoring were performed. Exercise was terminated immediately if participants experienced: (1) SpO₂ decrease to <85% or a drop of >4% from baseline; (2) heart rate exceeding 85% of age-predicted maximum; (3) significant dyspnea (Borg scale >6); (4) chest pain, severe dizziness, pallor, or diaphoresis; (5) onset of cyanosis or mental confusion (21). Emergency equipment including oxygen, resuscitation bag, and emergency medications were readily available in the rehabilitation area. All research nurses were trained in basic life support and emergency response protocols. Adverse events were documented on standardized forms, graded for severity (mild, moderate, severe), and reported to the principal investigator within 24 h. Serious adverse events were reported to the ethics committee within 7 days. A Data Safety Monitoring Board comprising an independent pulmonologist, cardiologist, and biostatistician reviewed safety data quarterly.

### 2.5 Outcome measures

Given that the ultimate goal of this multidimensional intervention is to improve health status, health outcomes were selected as the primary endpoints. These indicators represent the final common pathway through which all intermediate effects are ultimately manifested, thereby reflecting the clinical relevance of the intervention. All participants underwent measurements of four observed outcomes within 48 h of admission and at discharge to evaluate the effectiveness of the interventions, including forced expiratory volume in the first second (FEV1), 6-min walk test (6MWT), modified Medical Research Council dyspnea scale (mMRC-DS), and COPD Assessment Test (CAT).

#### 2.5.1 FEV1% predicted

FEV1 was tested again 8 weeks after discharge. It was measured for pulmonary ventilation function evaluation using a calibrated German Jaeger MasterScreen PFT System according to American Thoracic Society and European Respiratory Society standards (22). In our laboratory, test–retest reliability for FEV1 measurements is excellent, with intraclass correlation coefficient (ICC) of 0.98 (95%CI 0.96–0.99) based on 20 stable COPD patients tested 24 h apart. Inter-rater reliability between two trained technicians yielded ICC of 0.97 (95%CI 0.95–0.98). FEV1% predicted was calculated and used for analysis. It was interpreted according to the Global Initiative for Chronic Obstructive Lung Disease (GOLD) criteria: GOLD 1 (mild) defined as FEV1% ≥ 80% predicted, GOLD 2 (moderate) as 50% ≤ FEV1% < 80%, GOLD 3 (severe) as 30% ≤ FEV1% < 50%, and GOLD 4 (very severe) as FEV1% < 30% predicted (4). An absolute increase of 4–5% predicted or 100–140 mL is considered clinically meaningful (23).

#### 2.5.2 6MWT

Following ATS guidelines, the 6MWT was conducted in a flat and unobstructed 30-meter corridor, with participants’ heart rate, blood pressure, and blood oxygen saturation monitored (24). In our setting, test–retest reliability over 1 week in stable COPD patients (*n* = 20) demonstrated ICC of 0.94 (95%CI 0.90–0.97). The minimal clinically important difference (MCID) for 6MWT in COPD patients is reported as 25–30 meters (25). A distance <334 or <357 meters is associated with increased mortality and hospitalization risk in COPD patients (26).

#### 2.5.3 mMRC-DS

The mMRC-DS classifies the degrees of shortness of breath induced by daily activities into 5 grades: Grade 0 referring to only getting breathless with strenuous exercise, Grade 1 for getting short of breath when hurrying on level ground or walking up a slight hill, Grade 2 for walking more slowly than people of the same age on level ground because of breathlessness, or stopping for breath when walking at their own paces on the level ground, Grade 3 for stopping for breath after walking about 100 meters, or after a few minutes on level ground, Grade 4 for getting too breathless to leave the house, or getting breathless when dressing or undressing (27). Grade 0–1 indicates less breathlessness, while grade ≥2 indicates clinically significant dyspnea that limits daily activities and is associated with worse prognosis. It has demonstrated good construct validity when correlated with other dyspnea measures (*r* = 0.70–0.80) and predictive validity for mortality in COPD (28). Test–retest reliability over 2 weeks in our preliminary sample (*n* = 20) showed weighted kappa of 0.85 (95%CI 0.76–0.92), indicating excellent agreement.

#### 2.5.4 CAT

The CAT consists of eight items covering the most burdensome symptoms and limitations of COPD: cough, phlegm, chest tightness, breathlessness when going up hills/stairs, activity limitations at home, confidence leaving home, sleep, and energy (29). It has a scoring range of 0–40, with each item scoring 0 to 5 points based on the health impact of COPD. A total score of 0–10 points indicates a slight impact on life quality, 11–20 points for a medium impact, 21–30 points for a high impact, and 31–40 points for a very high impact. CAT scores were interpreted according to established categories: <10 indicates low impact, 10–20 indicates medium impact, 21–30 indicates high impact, and >30 indicates very high impact of COPD on health status. CAT has demonstrated strong internal consistency (Cronbach’s *α* = 0.88), test–retest reliability (ICC = 0.80), and construct validity against the St. George’s Respiratory Questionnaire (*r* = 0.80) in the original validation study (30). The MCID for CAT is reported as 2 points (31).

### 2.6 Statistical analysis

Statistical analyses were performed using Statistical Package for the Social Sciences (SPSS) Version 26. Prior to analysis, the normality of all continuous variables was assessed using the Shapiro–Wilk test and visual inspection of Q-Q plots. Variables with approximately normal distribution (Shapiro–Wilk *p* > 0.05) were presented as mean ± standard deviation. For variables that deviated from normality, non-parametric alternatives (Wilcoxon signed-rank test for within-group comparisons and Mann–Whitney U test for between-group comparisons) were planned; however, all primary outcome variables met normality assumptions, allowing the use of parametric tests. Baseline comparisons between groups and differences between pre- and post-intervention within groups were analyzed using Student’s t-test for independent samples and paired samples, respectively. Between-group comparisons of change scores (pre-post differences) were performed using independent samples t-tests. A two-tailed probability *p* < 0.05 was employed to indicate statistical significance.

## 3 Results

### 3.1 Baseline information

**Figure 1:**
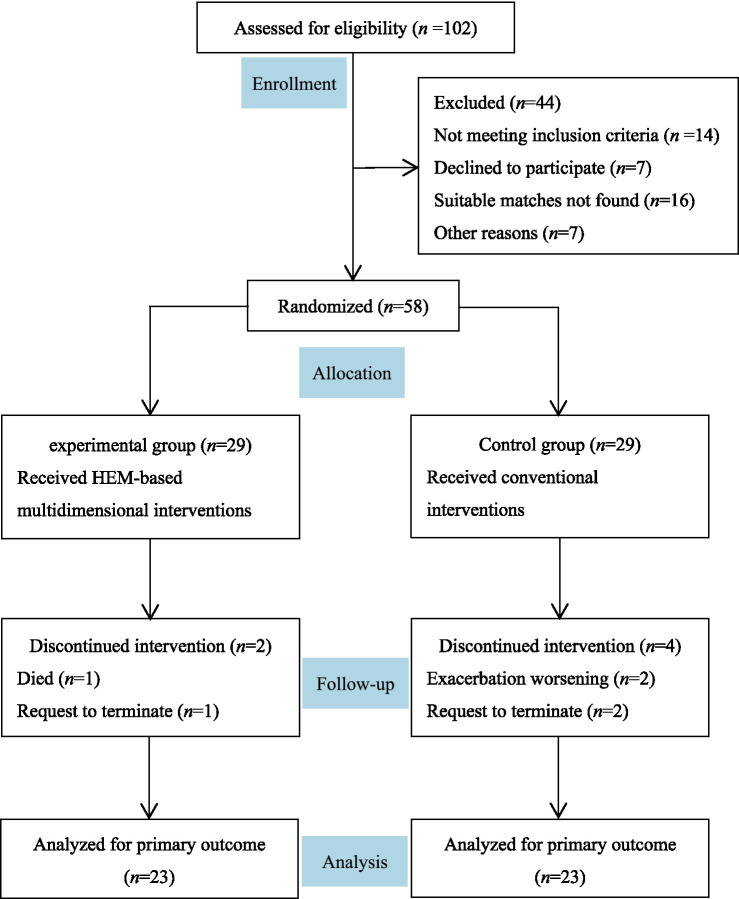
Flow chart of study implementation.

A total of 102 patients were assessed for eligibility, of whom 44 were excluded (14 did not meet inclusion criteria, 7 declined to participate, 16 could not be matched, and 7 were excluded for other reasons). The remaining 58 patients were randomized, and 46 completed the study (23 per group) as shown in Figure 1. There were 46 patients (namely, 23 pairs) with AECOPD who were ultimately included in the analysis (see Figure 1). Among them, 22 pairs were male, and 1 pair was female. Their ages ranged from 59 to 79 years, with an average of 69.30 years. The mean of COPD courses and inpatient days were 8.41 years and 7.96 days, respectively. The average BMI was 22.42 kg/m^2^, and 91.30% of the participants currently smoked. Twenty patients had a pulmonary function of Grade 3, while 10 had Grades 1 and 2, respectively. As shown in Table 1, there were no statistical differences in outcome variables (e.g., FEV1%, CAT) and potential confounding factors (e.g., age, hospital days) between the two groups at the baseline (all *p* > 0.05).

**Table 1:** Characteristics of participants at baseline by group.

Variable	Mean ± SD	*t*-value	*p*-value
Age (years)		−0.145	0.886
Experimental group	69.17±6.34		
Control group	69.44±5.89		
Body Mass Index		−0.245	0.808
Experimental group	22.26±4.78		
Control group	22.57±3.73		
Course of disease (years)		0.473	0.638
Experimental group	9.04±10.16		
Control group	7.78±7.75		
Hospital days		−0.132	0.896
Experimental group	7.91±2.45		
Control group	8.00±2.00		
FEV1%		0.097	0.923
Experimental group	50.28±19.46		
Control group	49.72±20.13		
CAT scores		0.869	0.390
Experimental group	20.83±3.92		
Control group	19.74±4.54		
mMRC scores		−0.986	0.330
Experimental group	2.48±0.73		
Control group	2.70±0.76		
6MWT (meters)		−0.958	0.343
Experimental group	364.24±75.91		
Control group	383.79±61.78		

### 3.2 Effectiveness evaluation based on self-control

As shown in Table 2, within-group analyses revealed significant improvements in all outcomes for the experimental group. The CAT scores decreased from 20.83 ± 3.92 to 9.91 ± 4.08 (mean reduction 10.92 points, 95%*CI* 9.64–12.20, *p* < 0.001), representing a 52.4% improvement and exceeding the minimal clinically important difference (MCID) of 2 points. Similarly, mMRC scores decreased from 2.48 ± 0.73 to 1.00 ± 0.52 (mean reduction 1.48 points, 95%*CI* 1.25–1.71, *p* < 0.001), a 59.7% reduction that surpasses the MCID of 1 point. The 6MWT distance increased by 50.77 meters (95%*CI* 34.13–67.41, *p* < 0.001), exceeding the MCID of 25–30 meters, and approached healthy endurance levels at 415.01 ± 72.51 meters post-intervention. FEV1% improved from 50.28 ± 19.46% to 57.00 ± 21.37% (mean increase 6.72, 95%*CI* 0.92–12.52, *p* = 0.025), exceeding the MCID of 4–5% and indicating a shift from severe to moderate obstruction classification.

**Table 2:** Comparison of the observed outcomes before and after intervention.

Variables	Before intervention		After intervention		*t*-value	*p*-value
	Mean ± SD	95%CI	Mean ± SD	95%CI		
CAT scores						
Experimental group	20.83 ± 3.92	19.13, 22.52	9.91 ± 4.08	8.15, 11.68	17.817	<0.001*
Control group	19.74 ± 4.54	17.77, 21.70	8.52 ± 2.63	7.39, 9.66	15.246	<0.001*
mMRC scores						
Experimental group	2.48 ± 0.73	2.16, 2.79	1.00 ± 0.52	0.77, 1.23	13.880	<0.001*
Control group	2.70 ± 0.76	2.37, 3.03	1.83 ± 0.59	1.58, 2.08	8.101	<0.001*
FEV1%						
Experimental group	50.28 ± 19.46	41.87, 58.70	57.00 ± 21.37	47.75, 66.24	−2.403	0.025*
Control group	49.72 ± 20.13	41.01, 58.42	49.70 ± 21.13	40.56, 58.84	0.017	0.987
6MWT (meters)						
Experimental group	364.24 ± 75.91	331.41, 397.06	415.01 ± 72.51	383.66, 446.37	−6.260	<0.001*
Control group	383.79 ± 61.78	357.08, 410.51	419.16 ± 65.00	391.05, 447.27	−4.873	<0.001*

^*^p-value <0.05.

In the control group, significant within-group improvements were also observed for CAT scores (mean reduction 11.22 ± 3.53 points, *p* < 0.001), mMRC scores (mean reduction 0.87 ± 0.59 points, p < 0.001), and 6MWT distance (mean increase 35.37 ± 34.81 meters, *p* < 0.001). However, FEV1% remained essentially unchanged (mean change −0.02 ± 5.15%, *p* = 0.987). When compared against MCID thresholds, the control group achieved clinically meaningful improvements in CAT (11.22 points >2) and 6MWT (35.37 meters >30), but not in mMRC (0.87 points <1) or FEV1% (−0.02% < 4–5%). These findings demonstrate that while both groups achieved clinically significant gains in quality of life and exercise capacity, only the experimental group achieved clinically meaningful improvements in dyspnea and lung function.

### 3.3 Effectiveness evaluation based on parallel control

As displayed in Table 3, significant between-group differences favoring the experimental group were observed for mMRC score reduction (mean difference −0.61 points, 95%CI -0.88 to −0.26, *p* = 0.001) and FEV1% improvement (mean difference 6.74, 95%CI 0.61–12.87, *p* = 0.032). These differences exceeded the MCID for mMRC (1 point) and approached the MCID for FEV1% (4–5%), suggesting clinically meaningful additive benefits of the HEM-based intervention for dyspnea relief and lung function. However, no significant between-group differences were detected for CAT score reduction (mean difference −0.30 points, 95%CI -2.25 to 1.63, *p* = 0.752) or 6MWT increase (mean difference 15.41 meters, 95%CI -6.44 to 37.26, *p* = 0.164), despite both groups demonstrating substantial within-group improvements (Table 2).

**Table 3:** Comparison of the observed outcome changes before and after the interventions.

Variables	Experimental group		Control group		*t*-value	*p*-value
	Mean ± SD	95%CI	Mean ± SD	95%CI		
Decrease of CAT scores	10.92 ± 2.94	9.64, 12.18	11.22 ± 3.53	9.69, 12.74	0.318	0.752
Decrease of mMRC scores	1.48 ± 0.51	1.26, 1.70	0.87 ± 0.53	0.63, 1.11	−3.681	0.001*
Increase of FEV1%	6.72 ± 13.40	0.92, 12.51	−0.02 ± 5.15	−2.24, 2.21	2.249	0.032*
Increase of 6MWT (meters)	50.78 ± 38.90	33.95, 67.60	35.37 ± 34.81	20.32, 50.42	1.415	0.164

^*^p-value <0.05.

## 4 Discussion

Building on the within-group and between-group findings presented in the results, this paired interventional study demonstrates that the multidimensional HEM-based PR program achieved statistically significant improvements in all outcomes within the experimental group. However, when comparing the incremental benefit over conventional care, only improvements in mMRC and FEV1% reached statistical significance, with the FEV1% improvement also meeting criteria for clinical significance (exceeding MCID). These findings highlight the importance of distinguishing between within-group changes (which reflect overall recovery) and between-group differences (which isolate the specific contribution of the HEM components). The conventional regimen, encompassing health education, oxygen therapy, as well as PLB and DB exercises, was also demonstrated to be safe and to provide some therapeutic benefit during AECOPD.

Previous studies have shown that early PR during or after an acute exacerbation of COPD is safe and feasible, and earlier implementation may lead to faster improvement in bodily performance (32, 33). An RCT conducted on patients with AECOPD and community-acquired pneumonia (CAP) further strengthened such a conclusion, proposing that the gains of physical therapy intervention exceed the deterioration caused by immobilization during hospitalization (34). Another study employing similar interventions and metrics to those in our study proposed that early initiation of PR resulted in significant improvements in patient-reported symptoms and 6MWT outcomes, without an extended hospital stay (35). Both the timing and outcomes of PR, therefore, are proactive and should not be obstacles for PR execution. Moreover, low-flow oxygen inhalation benefits the ventilatory function of patients with COPD by increasing oxyhemoglobin saturation and preventing carbon dioxide retention, and the combination of PLB and DB exercises is widely recognized as an easy and low-cost physical therapy intervention (36, 37). As a result, the joint application of oxygen therapy, breathing exercises, and standardized health education contributed to the improvement of dyspnea and quality of life. The 6-min walking distance markedly increased to 419 meters, with a rise of 36 meters, which was 15 and 26 meters less than that of our experimental group and that reported by Gloeckl R et al., respectively (38). Similar to a study involving moderate-intensity aerobic exercise for PR, the FEV1% remained almost unchanged, nevertheless (39). Conventional interventions appear to be ineffective in improving breathing obstruction for the present.

The host, agent, and environment jointly affect the occurrence of infectious diseases, according to the triangle model proposed by American epidemiologist John E. Gordon in the mid-20th century. HEM uniformly highlights the roles of environment and further classifies individual factors into physiological and psychological dimensions, offering a more comprehensive perspective for non-infectious chronic disease (NCD) management. The strong social nature of human beings also calls for multifaceted and long-term actions in addition to medical care. A meta-analysis that aggregated the results of 13 RCTs indicated that PR alone did not lead to an increase in FEV1 (40). Patel S et al. suggested that engagement of informal carers and support from peers may help maximize the utilization of PR (41). As a result, this study precisely incorporates the HEM concept and strategy of multidimensional intervention for PR, achieving positive results. As part of the HEM-based intervention, medical insurance staff were invited to provide policy education to patients, addressing potential financial barriers to rehabilitation engagement. Financial concerns have been identified as a significant barrier to PR completion in COPD patients, particularly in low- and middle-income settings (16). By enhancing patients’ understanding of available insurance coverage and reimbursement policies, this component aimed to alleviate economic stress and potentially improve adherence—a factor associated with better rehabilitation outcomes (42). While we observed significant improvements in several clinical outcomes, the specific contribution of the insurance education component cannot be isolated from the multidimensional intervention. Future studies should examine the independent effect of financial counseling on rehabilitation adherence and clinical outcomes. Given the well-documented association between meteorological factors and COPD symptoms, the HEM-based intervention included regulation of indoor temperature and humidity in patients’ wards and homes (43). While we observed significant improvements in FEV1% and mMRC scores in the experimental group, we cannot attribute these gains specifically to the environmental regulation component. Objective measures of environmental factors—such as indoor air quality (PM2.5, PM10), precise ventilation rates, or consistent humidity logging—were not assessed in this study. Therefore, the independent contribution of environmental regulation to the observed clinical improvements remains unclear. The temperature and humidity targets applied in this study (22–25 °C in winter, 23–28 °C in summer; 30–80% humidity in winter, 40–60% in summer) were based on general comfort guidelines rather than evidence-based thresholds for respiratory outcomes. Future studies should incorporate objective environmental monitoring to examine dose–response relationships between indoor climate parameters and pulmonary function.

The significant improvements observed in the experimental group for FEV1% and mMRC scores—outcomes that typically show limited response to conventional PR alone—suggest that the HEM-based multidimensional intervention may offer benefits beyond conventional care for these specific outcomes. However, the mechanisms underlying these improvements cannot be definitively determined from the current data. While we hypothesize that the psychological components (peer and caregiver support) and environmental regulation may have contributed to enhanced adherence and self-efficacy—factors known to influence rehabilitation outcomes—these mediating variables were not directly measured in this study (41). Therefore, any attribution of causality to specific intervention components remains speculative. Future studies should incorporate validated measures of potential mediators such as exercise adherence, self-efficacy, social support, and psychological well-being to elucidate the pathways through which HEM-based interventions exert their effects. Despite this, the changes in CAT and 6MWT do not present significance. The lack of significant differences in CAT scores and 6MWT may be explained by the higher proportion of severe COPD cases, advanced age of participants, and relatively limited intervention intensity and duration.

Several limitations should be acknowledged. The single-center design and modest sample size (N = 46) limit generalizability, and the 1:1 matching excluded 16 patients, potentially introducing selection bias. Although outcome assessors were blinded, the open-label intervention delivery may have introduced performance bias. The multidimensional nature of the HEM-based intervention precludes identification of which specific components drove the observed improvements, and mechanistic insights are limited by the absence of validated mediator measures (e.g., self-efficacy, adherence, social support). Objective environmental monitoring was not performed, preventing verification of intervention fidelity. The 8-week follow-up is insufficient to assess long-term sustainability, and the predominance of male participants (95.7%) limits applicability to female patients. Future research should address these limitations through multi-center trials with larger, diverse samples, longer follow-up periods assessing hard clinical endpoints (exacerbations, readmissions, mortality), and inclusion of validated mediator measures to elucidate mechanisms. Objective environmental monitoring and factorial or dismantling designs are needed to isolate the independent effects of specific HEM components. Cost-effectiveness analyses and subgroup studies are also warranted to inform targeted implementation.

## 5 Conclusion

This preliminary study demonstrates that a multidimensional pulmonary rehabilitation program based on the Health Ecology Model, when added to conventional care, yields clinically meaningful improvements in lung function and dyspnea in patients hospitalized with AECOPD. These findings support the integration of multi-level interventions—including physiological training, psychological support, and environmental regulation—into standard care for optimizing outcomes in domains that show limited response to conventional rehabilitation. Further research with mechanistic measures and longer follow-up is warranted to confirm these observations.

## Data Availability

The datasets presented in this article are not readily available because the patient provided informed consent, agreeing that the data collected in this study will be withheld from third parties, including in anonymized form. Requests to access the datasets should be directed to Huamei Zheng, 13556175767@163.com.
